# Synthesis of metal organic framework-based hydrogel for microplastics removal from aquatic environments

**DOI:** 10.1007/s11356-026-38156-2

**Published:** 2026-09-01

**Authors:** Thi Trang Luu, Shudi Mao, Dai Quyet Truong, Yihan Shi, Thi Ngoc Oanh Duong, Qiang Fu, Van Manh Do, Saravanamuthu Vigneswaran, Tien Vinh Nguyen

**Affiliations:** 1 Faculty of Engineering and IT, University of Technology Sydney (UTS), Sydney, NSW 2007 Australia; 2 Faculty of Urban Infrastructure and Environment Engineering, Hanoi Architectural University, Hanoi, 100000 Vietnam; 3 Institute of Science and Technology for Energy and Environment, Vietnam Academy of Science and Technology (VAST), Hanoi, 100000 Vietnam; 4 Central Institute for Natural Resources and Environmental Studies, Vietnam National University, Hanoi, 100000 Vietnam

**Keywords:** Metal organic framework, Composite hydrogel, Microplastic removal, Filtration

## Abstract

Microplastics (MPs) pollution has emerged as a pressing global environmental problem. Current research in this field remains exploratory because there is a lack of standards, methods, and policies for the detection, removal, and regulation of MPs discharge. In this study, a series of metal–organic framework (MOFs)-incorporated hydrogels was successfully fabricated via a photo-initiated cross-linking strategy, offering a facile and efficient preparation approach applicable to the removal of diverse MPs types. Among them, ZIF–8.1@H6mg exhibited the highest MPs removal efficiencies, achieving up to 97% for polyethylene terephthalate (PET) and 98% for polyethylene (PE) in laundry wastewater, one of the main sources of MPs pollution. The material demonstrated excellent recyclability, with MPs removal efficiency only slightly decreasing to 96% after six reuse cycles. It maintained stable operation for approximately 4 h, and pre-treatment with backwashing further enhanced long-term filtration performance. Comprehensive characterization of the ZIF-8 hydrogel composites suggests that their interpenetrating network structures, zeta potential, and surface functional groups contributed to their superior performance. These findings highlight the potential of ZIF-based hydrogels as sustainable and effective materials for mitigating the growing threat of MPs pollution.

**Supplementary Information:**

The online version contains supplementary material available at https://doi.org/10.1007/s11356-026-38156-2.

## Introduction

Microplastics pollution is currently a serious global issue. The term *microplastics* (MPs) was first introduced by Thompson et al. (2004), who defined them as plastic particles smaller than 5 mm, although the majority are actually less than 500 μm in size (Thompson et al. 2004). They are composed of 14 different polymers and occur in various shapes, most commonly as fibers, fragments, and films (Wang et al. 2020). The main wastewater sources with high MPs concentrations include laundry effluent, wastewater from plastic recycling processes, leachate from landfills, and sludge from wastewater treatment plants (De Falco et al. 2018; Stapleton et al. 2023; Nguyen et al. 2022; He et al. 2019). Microplastics have emerged as dangerous contaminants due to their persistence, widespread distribution, and potential adverse impacts on aquatic organisms and human health (Ansari et al. 2025). Because MPs are associated with chemicals from production and manufacturing processes, and they can also adsorb substances from the surrounding environment, their ingestion can cause harmful effects on both wildlife and humans through combined physical and chemical toxicities (Prata et al. 2020; Smith et al. 2018). It is therefore deemed essential to develop effective methods for removing MPs from the aquatic environment. Recent studies indicate that physicochemical methods can effectively remove MPs under appropriate environmental conditions. Among them, flocculation, adsorption, sand filtration, and flotation are relatively low-cost techniques, whereas membrane-based approaches are more expensive. Notably, membranes offer the distinct advantage of achieving very high removal efficiencies across all MPs size ranges, particularly when integrated with other treatment processes (Luu et al. 2025). By contrast, the effectiveness of flotation and sand filtration is strongly influenced by particle size. The primary limitation of flotation lies in its operational complexity, while coagulation is constrained by challenges associated with residual management (Tang et al. 2021, Amirah Mohd Napi et al. 2023). Among the available treatment technologies, adsorption has attracted increasing attention due to its operational simplicity, low energy requirements, and a high removal efficiency for a wide range of contaminants, including MPs (Rai et al. 2026).

Recently, metal–organic frameworks (MOFs) have emerged as highly effective materials for addressing pollution in aquatic environments due to their exceptional porosity, extensive surface area, and strong selectivity toward pollutants (Kitaura et al. 2002; Li et al. 1999; Keshta et al. 2026; Arora et al. 2025; Dutta et al. 2022). In aquatic environments, MOFs containing positively charged ions are well-suited for MPs removal, as most MPs acquire negative surface charges in water, enabling strong electrostatic interactions that promote effective capture. However, several recent studies have reported relatively low removal efficiencies, achieving only about 32.6% (Gao et al. 2023) or 76.5% (Qin et al. 2024). Barari et al. (2025) evaluated the performance of MOFs in MPs removal by investigating findings from more than 65 studies and proposed that future research should aim to improve synthesis efficiency, scale up MOFs applications, and assess their environmental implications (Barari et al. 2025). The reason is that MOFs materials are typically in powdered form. At the macroscopic scale, pristine MOF powders typically exist as fine and discrete particles, which limit their structural stability and hinder their practical deployment in continuous water treatment systems. This intrinsic limitation restricts the scalability of MOF-based adsorption technologies despite their promising affinity toward MPs. Therefore, developing strategies to structurally integrate MOFs into robust, macroscopic architectures is essential for translating their intrinsic adsorption capability into practical applications.

Hydrogels have recently emerged as an attractive platform for constructing such composite architectures, owing to their three-dimensional porous networks, high water permeability, and tunable mechanical properties (Mao et al. 2025, 2023). By embedding MOFs within a hydrogel matrix, it becomes possible to combine the high surface activity of MOFs with the structural integrity and processability of polymeric scaffolds.

This research focuses on developing a novel composite hydrogel absorption platform composed of MOF-fillers and an interpenetrating hydrogel matrix for removing MPs from aquatic environments. Firstly, an optimal zeolitic imidazolate framework (ZIF-8) was synthesized through a facile method then ZIF-8 was incorporated into a double network hydrogel matrix to produce a new MOF-based hydrogel material via the UV-initiated cross-linking method. Here, the hydrogel material was selected since it exhibits well-defined porous architectures, low-cost, high-energy efficiency, and simple preparation (Han et al. 2025; Feng et al. 2024). This new composite material is environmentally friendly, reusable, and scalable for large-scale applications. Secondly, the performance and mechanism of a new composite material for removing polyethylene (PE) and polyethylene terephthalate (PET), two of the most common types of MPs, were investigated through a low-pressure filtration column, enabling simple, continuous, and efficient operation.

## Experimental

### Materials

2-Methylimidazole (HMIM, 99%), zinc nitrate hexahydrate (Zn(NO₃)₂·6H₂O, 98%), polyvinyl alcohol (PVA, molecular weight 89,000–98,000 g/mol), poly(ethylene glycol) diacrylate (PEGDA, molecular weight 575 g/mol), lithium phenyl-2,4,6-trimethylbenzoylphosphinate (LAP,), glutaraldehyde solution (GA, 25 wt%), hydrochloric acid (HCl, 32%), polyethylene (PE 300), polyethylene terephthalate (PET 300), hydrogen peroxide (H₂O₂, 30% in water), and aluminium oxide filters (Anodisc, 0.2 μm pore size, 25 mm diameter, Whatman) were all purchased from Merck and used without further purification.

### Synthesis of zeolitic imidazolate framework, ZIF—8

The ZIF-8 nanomaterial was synthesized using a conventional solvothermal method (Pan et al. 2011). Specifically, 250 mL and 125 mL of 1.2 M 2-methylimidazole (HMIM) solutions were separately mixed with 100 mL of 0.5 M zinc nitrate hexahydrate (Zn(NO₃)₂·6H₂O) solution in glass beakers to obtain ZIF—8.1 and ZIF—8.2, respectively. The mixtures were stirred with a magnetic stirrer for 1 h, followed by centrifugation. The precipitates were washed once with deionized (DI) water and twice with ethanol to remove residual organic ligands. Finally, the resulting white powders of ZIF—8.1 and ZIF—8.2 were collected and dried overnight at 85 °C in a convection oven (see schema 1 A).

### Synthesis of zeolitic imidazolate framework-based hydrogel, ZIF – 8.1 @H6mg

**Scheme 1:**
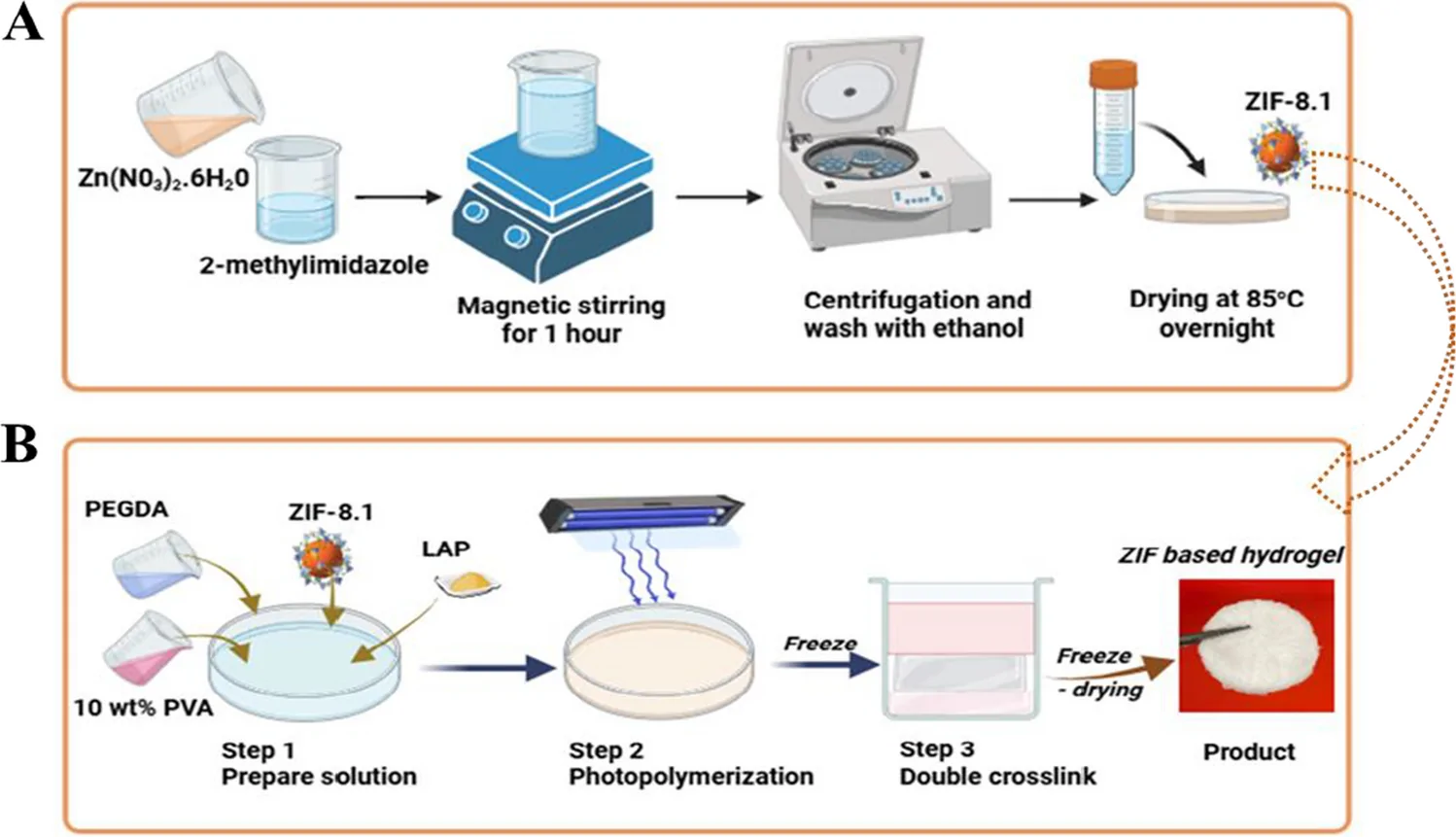
Illustration of zeolitic imidazolate framework-based hydrogel. (**A**) Step-by-step procedure for ZIF—8.1, ZIF – 8.2; (**B**) Procedure for ZIF based hydrogels (Created in BioRender. https://BioRender.com/74q0k37)

A 10 wt% PVA solution was prepared by dissolving 10 g of PVA powder in 100 mL of DI water at 90 °C under continuous stirring for 5 h. To formulate the precursor solution, 6 mg of ZIF—8 was firstly dispersed in 2 mL of DI water via ultrasonication for 5 min to achieve uniform dispersion. Subsequently, 150 mg of PEGDA, 3 mg of LAP, and 1 mL of the prepared 10 wt% PVA solution were added sequentially, followed by thorough stirring to obtain a homogeneous mixture. The resulting solution was cast into a transparent mould and subjected to UV irradiation (λ_max_ = 390 nm, 60 W) for 10 min to complete the photopolymerization process. The formed hydrogel was then frozen at –20 °C overnight. To chemically cross-link the PVA chains, the frozen ZIF-based hydrogel was immersed in a solution containing 0.375 wt% GA and 0.08 wt% HCl for 30 min, and this treatment was repeated. Finally, the ZIF-based hydrogel was thoroughly rinsed with DI water to remove any residual chemicals and subsequently freeze-dried prior to the filtration tests (see scheme 1B). Two ZIF-8 materials (ZIF-8.1 and ZIF-8.2) were synthesized and characterized. Based on the characterization results, ZIF-8.1 was selected for hydrogel fabrication due to its superior crystallinity and porous structure. Consequently, only ZIF-8.1 was incorporated into the hydrogel composites (H3mg, H6mg, and H9mg) (Table 1).

**Table 1:** Composition of the mixture used in this study

Label	Zn(NO_3_)_2_.6H_2_O (0.5 M) (mL)	HMIM (0.2 M) (mL)	ZIF-8.1 loading in hydrogel(mg per 100 mL)
ZIF—8.1	100	250	
ZIF—8.2	100	125	
ZIF—8.1@H3mg			3
ZIF—8.1@H6mg			6
ZIF—8.1@H9mg			9

### Continuous-flow column filtration experiment

**Fig. 1:**
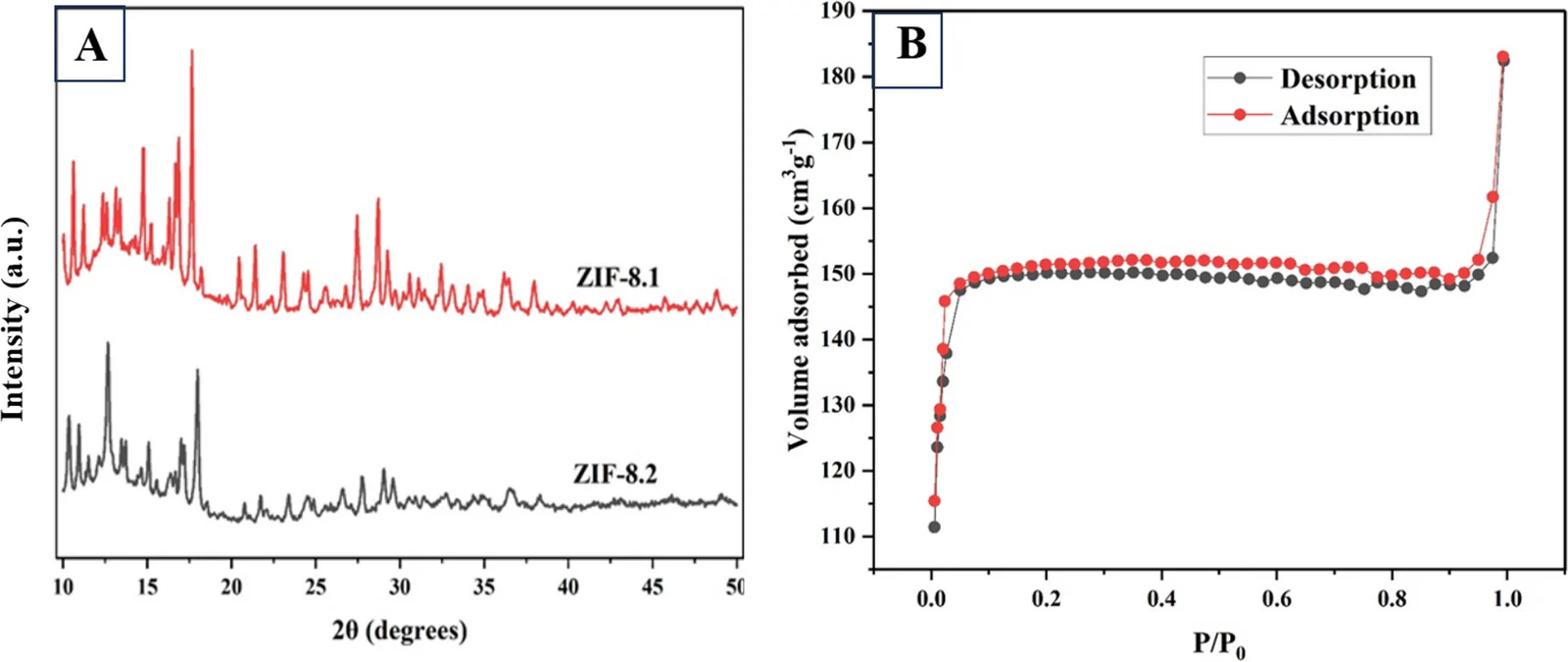
(**A**) The XRD pattern of ZIF – 8.1 and ZIF – 8.2. (**B**). The isotherm characteristics of ZIF-8.1

**Fig. 2:**
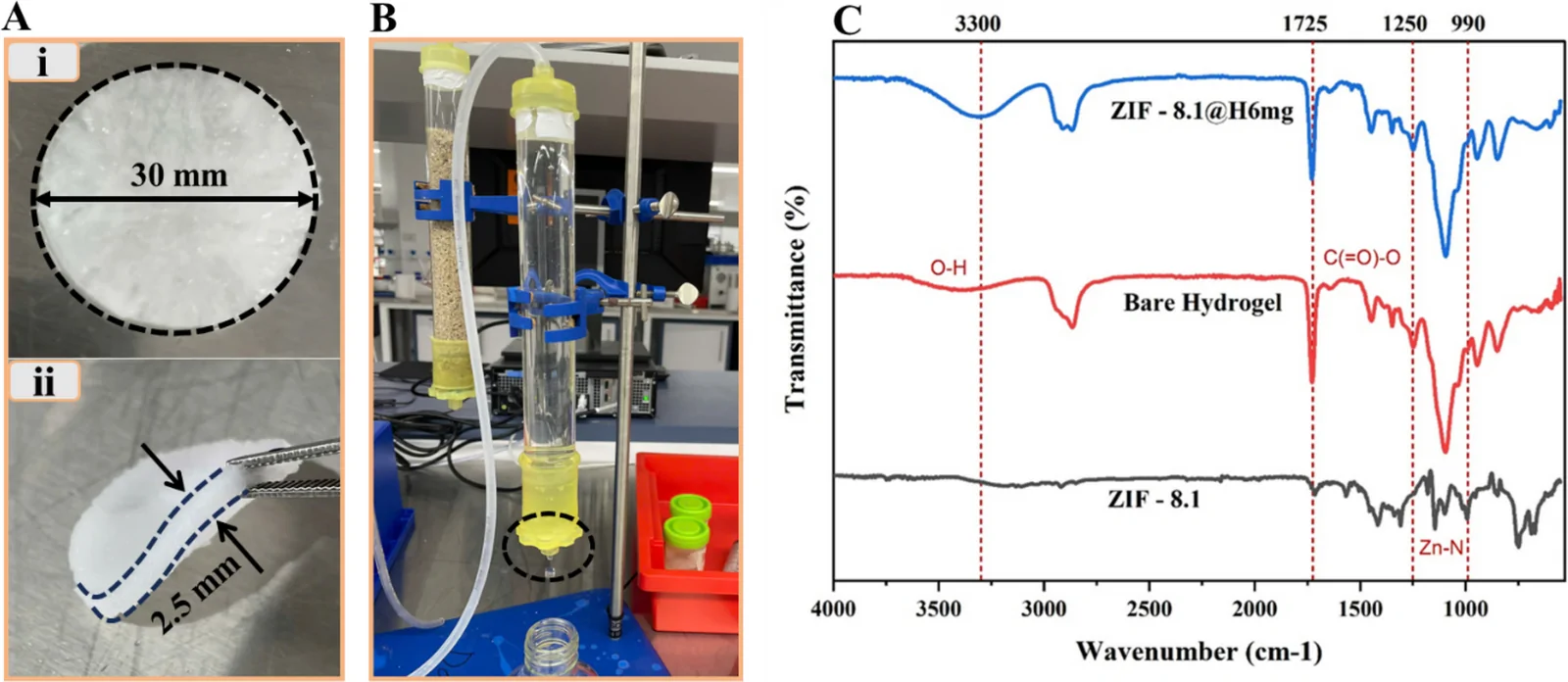
(**A**) Optic image of ZIF-based hydrogel (**B**). Image of the filtration column system (**C**) FT-IR spectra of ZIF-based hydrogel

The ZIF-incorporated (Fig. 1) hydrogel materials were washed with deionized (DI) water and subsequently freeze-dried prior to characterization and MPs filtration tests. For the continuous-flow filtration experiment, a ZIF-based hydrogel sample with a 30 mm diameter (Fig. 2A) was fabricated to ensure a tight fit at the bottom of the filtration column. The column had an inner diameter of 30 mm and a height of 280 mm (Fig. 2B). The thickness of the hydrogel filter was 2.5 mm. A flow rate of 1.2 L h⁻^1^, corresponding to a filtration rate of 1.7 m h⁻^1^, was applied throughout the column experiments. The filtration system served to evaluate MPs removal performance, filtration stability, and material reusability under continuous-flow conditions. An ADAM DU data acquisition system (Advantech, Taiwan) was deployed to continuously record filtration rate during the experiments. The recorded data were subsequently analyzed to quantitatively assess fouling behavior and filtration performance under different operating conditions.

## Preparation of laundry wastewater

Real laundry wastewater samples were obtained from a residential washing machine (Bosch Serie 4 WLG24225) following two wash/spin cycles. The washing load consisted of 5 kg of clothes, with the T-shirts containing 65% synthetic fibers and 35% cotton. A detergent dosage of 45 mL per washing cycle was used. In addition, the number and types of garments were kept constant throughout all experiments to ensure consistency and comparability among different sampling events. Washing was conducted using the synthetic clothes program at 40 °C and 1200 rpm in a 30-min quick wash mode. Wastewater was collected directly from the machine’s drainpipe after two rinsing steps, specifically at 12 and 18 min into operation. The sampling times of 12 and 18 min correspond to the beginning of the two main rinsing cycles of the washing program. Water samples were collected continuously for 5 min during each rinsing cycle. The wastewater collected from both rinsing stages was subsequently combined to: firstly, obtain a representative sample of the overall laundry effluent; and secondly, minimise potential variations in wastewater characteristics between individual rinsing cycles (Fig. S1).

To obtain a wastewater matrix with elevated MPs concentrations, particles from two very common MPs types, polyethylene (PE 300) and polyethylene terephthalate (PET 300), were spiked into the raw household laundry effluent. This adjustment produced solutions containing 100 mg L^−1^ of each polymer. The added PE and PET particles ranged from approximately 20 to 1000 µm in size, with respective densities of 0.91 g cm^−3^ and 1.3 g cm^−3^. The elevated MPs concentrations were intentionally selected to standardize the influent MPs loading among experiments, thereby improving the accuracy and reproducibility of removal efficiency measurements. Furthermore, considering the large variability in MPs concentrations reported across different wastewater sources, these conditions were adopted as a proof-of-concept approach so that the treatment performance under relatively challenging conditions could be evaluated.

Regarding MPs identification, a pre-determined volume of wastewater samples (50 mL) was mixed with an equal volume of 30% H₂O₂ to remove organic matter and digested at 100 °C for 2 h. The resulting MP-containing solution was filtered through a 0.2 µm Anodisc filter using a vacuum pump, and the filters with retained MPs were stored in covered glass Petri dishes for further analysis (Masura et al. 2015).

### Characterization methods

A PerkinElmer Spectrum 3 FTIR spectrometer equipped with a Spotlight 200i microscope (PerkinElmer, USA) was used for the identification and quantification of MPs (Tarte et al. 2024). The analyses were conducted using Spectrum IR software (PerkinElmer, USA), with PerkinElmer spectral libraries applied as reference databases (Fig.S2). Fourier transform infrared spectroscopy (FTIR, MIRacle 10, Shimadzu) analyses were conducted over a scan range of 4000–600 cm⁻^1^ to examine the chemical composition and functional groups of the synthesized ZIF-8-based hydrogel materials before and after filtration. The efficiency in removing MPs was determined based on the reduction in particle counts after treatment compared with the particle counts measured in the influent of the corresponding treatment process, as expressed in Eq. (1).

1 $${\mathrm{E}}_{\mathrm{r}\mathrm{e}\mathrm{m}\mathrm{o}\mathrm{v}\mathrm{a}\mathrm{l}} = (1-{\mathrm{C}}_{\mathrm{M}\mathrm{P}\mathrm{s}\_\mathrm{e}\mathrm{f}\mathrm{f}\mathrm{l}\mathrm{u}\mathrm{e}\mathrm{n}\mathrm{t}} / {\mathrm{C}}_{\mathrm{M}\mathrm{P}\mathrm{s}\_\mathrm{i}\mathrm{n}\mathrm{f}\mathrm{l}\mathrm{u}\mathrm{e}\mathrm{n}\mathrm{t}}) \mathrm{x} 100\mathrm{\%}$$

where C_MPs_influent_ is the concentration of MPs (particles L⁻^1^) in the influent before treatment, and C_MPs_effluent_ is the concentration of MPs (particles L⁻^1^) after treatment.

Nitrogen adsorption – desorption measurements of ZIF-8.1 and ZIF-8.2 were executed using a BET surface area analyzer with nitrogen as the adsorbate (Micromeritics TriStar II Plus, Australia). The specific surface area (S_BET) was determined using the standard Brunauer–Emmett–Teller (BET) method. The total pore volume (V_total_) was estimated from the amount of liquid nitrogen adsorbed at a relative pressure (P/P₀) of approximately 0.99 (Quyet Truong et al. 2023). Zeta potential analysis was performed to evaluate the surface charge of the MPs and ZIF-8.1 and ZIF-8.2 nanomaterials. Measurements were conducted using a Zetasizer Nano instrument (Malvern, UK).

The surface morphology of the ZIF-8-based hydrogels was examined using scanning electron microscopy (SEM; Zeiss Supra 55VP) at an acceleration voltage of 10 kV, both before and after the MPs removal process. Oxford energy dispersive spectroscopy (EDS) mapping was used for the ZIF-8.1@H6mg sample to analyse and identify the distribution of elemental components within the material. The crystalline structures of the ZIF-8-based hydrogels were characterized using X-ray diffraction (XRD; Bruker D8 Discover) with Cu Kα radiation. Powdered samples were scanned at a step size of 0.04°/s over a 2θ range of 5° to 50°.

## Quality control

In each experiment, at least three sets of MPs concentration data were collected to calculate average values. All glassware was first cleaned in a laboratory-grade washing machine, then rinsed with 90% ethanol and wiped with antistatic cloths prior to use. Between samples, the glass filtration funnel was rinsed with ethanol and deionized water. Glass equipment, including Petri dishes and containers, was used wherever possible to minimize MPs contamination from laboratory materials.

## Results and discussion

### The synthesis of composite hydrogel absorbents

In this study, a novel hydrogel absorbent composed of MOF fillers embedded within a cross-linked polymer matrix was developed. Firstly, the obtained ZIF-8 fillers were characterized by XRD and BET analyses, and the results are presented in Fig. 1.

A quantitative analysis of the XRD data was undertaken and is presented in Supplementary Table S1 and Fig. S3. ZIF-8.1 exhibited a higher maximum diffraction peak intensity (18.38 a.u.) than ZIF-8.2 (12.73 a.u.), corresponding to an increase of approximately 44%. Furthermore, the full width at half maximum (FWHM) of the major diffraction peak for ZIF-8.1 was 0.18°, which was substantially lower than that of ZIF-8.2 (0.42°). The higher peak intensity and narrower FWHM indicate sharper diffraction peaks and a higher degree of crystallinity for ZIF-8.1. These results are consistent with the XRD patterns shown in Fig. 1A and support the selection of ZIF-8.1 for subsequent hydrogel fabrication. Specific surface analysis (BET) was subsequently conducted on the ZIF-8.1 sample to evaluate its textural properties. The isotherm characteristics of ZIF-8.1 demonstrate a typical behavior of microporous materials (Fig. 1B). A steep uptake is observed at very low relative pressures (P/P₀ < 0.01), indicating rapid adsorption into micropores with pore sizes less than 2 nm from the outset. This is followed by early saturation and the formation of a plateau, as the micropores become fully occupied and no further increase in adsorption occurs despite increasing pressure.

Additionally, the isotherm exhibited no obvious hysteresis loop, indicating a predominantly microporous structure and the absence of significant mesoporous or macroporous pores, where capillary condensation typically generates hysteresis behavior (Thommes et al. 2015). As a result, capillary condensation—commonly associated with mesopores—is not observed in ZIF-8. Furthermore, the surface area determined by the Density Functional Theory for adsorption isotherm analysis (DFT method) was 496.421 m^2^/g, which is consistent with previous studies (Quyet Truong et al. 2023; Chen et al. 2020). Overall, both the XRD pattern and the isotherm profile shown in the graphs confirm that ZIF-8.1 material has been successfully synthesized with well-developed microporous characteristics.

The FTIR spectra of the ZIF-8, bare PVA/PPEGDA hydrogel, and the ZIF-8.1@H6mg composite hydrogel are shown in Fig. 2C. Regarding the ZIF-8.1, peaks in the 1580–1500 cm⁻^1^ range were assigned to C = N or C = C stretching within the imidazole ring, and signals between 1350 and 900 cm⁻^1^ were associated with Zn–N stretching and imidazole ring vibrations (Ahmad et al. 2024; Mittal et al. 2022). In the case of the ZIF—8.1@H6mg composite, where 6 mg of ZIF-8 was incorporated into the hydrogel matrix, new peaks appeared in the 1500–1350 cm⁻^1^ and 800–900 cm⁻^1^ regions, confirming the successful integration of ZIF – 8.1. While some hydrogel-specific peaks (e.g., C = O- and ether group) were still present, their reduced intensities suggest possible coverage or interactions between the ZIF – 8.1 particles and the hydrogel network. These spectral features collectively confirm the formation of a composite material that retains the characteristics of both the hydrogel and ZIF-8 components. The infrared spectroscopy was used to demonstrate the intramolecular and intermolecular interactions of ZIF-8 and the hydrogel (Fig. 2C). The FTIR spectrum of the bare hydrogel showed a relatively flat profile with several distinguishable characteristic peaks. A broad band around 3300 cm⁻^1^ corresponded to O–H stretching vibrations, likely arising from hydroxyl groups or adsorbed water. A peak at 1725 cm⁻^1^ can be attributed to the asymmetric stretching of carbonyl groups (C = O-), while the region 1102 cm⁻^1^ was associated with C–O–C stretching vibrations, characteristic of polysaccharide structures (Sener Raman et al. 2022). These features indicate the presence of hydroxyl functional groups.

**Fig. 3:**
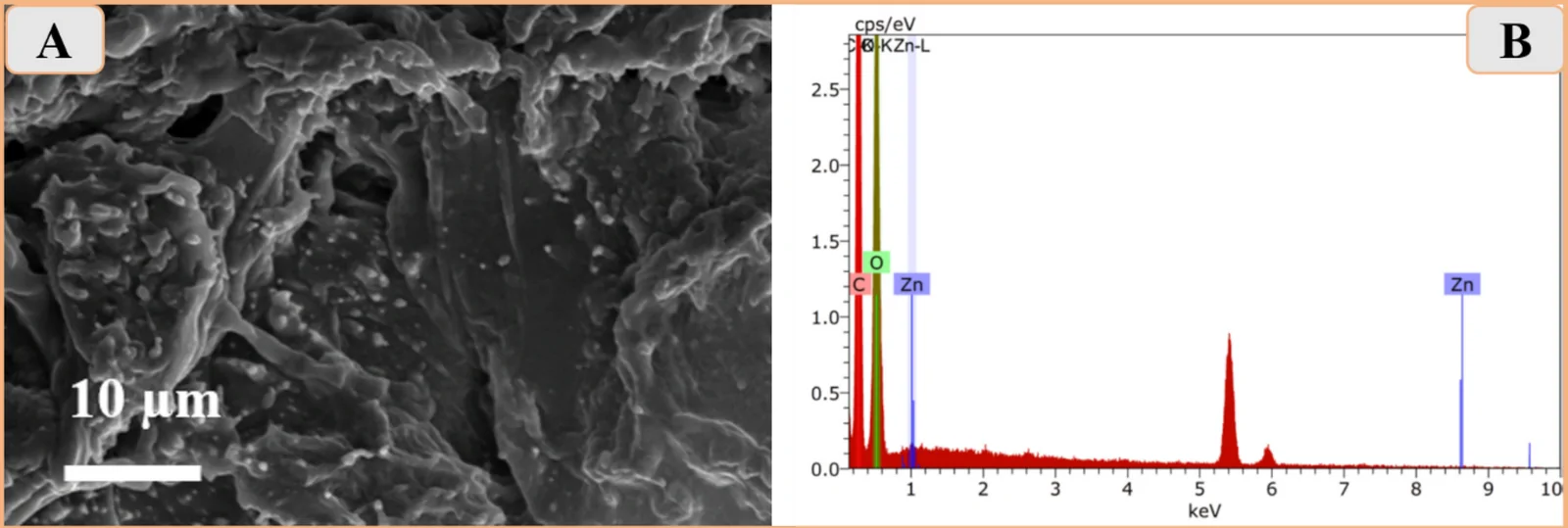
A(**A**) SEM images of ZIF – 8.1@H6mg, (**B**) Elemental mapping of ZIF – 8.1@H6mg

Furthermore, scanning electron microscopy (SEM) images revealed the presence of small crystalline particles attributed to ZIF-8.1 on the hydrogel skeleton, indicating successful incorporation of ZIF-8.1 into the hydrogel matrix (Fig. 3A). The EDS spectrum of ZIF-8.1@H6mg was dominated by signals from carbon and oxygen, which are the major constituent elements of the PVA/PEGDA hydrogel matrix. In contrast, the Zn signal was relatively weak (Fig. 3B and S6). Instead of attributing this observation solely to the encapsulation of ZIF-8 particles within the hydrogel network, several factors may contribute to the reduced Zn intensity. These include the relatively low ZIF-8 loading in the composite, the predominance of the polymer-rich matrix, and the limited sensitivity of SEM–EDS for detecting nanoparticles embedded beneath the sample surface.

### Microplastics removal performance

**Fig. 4:**
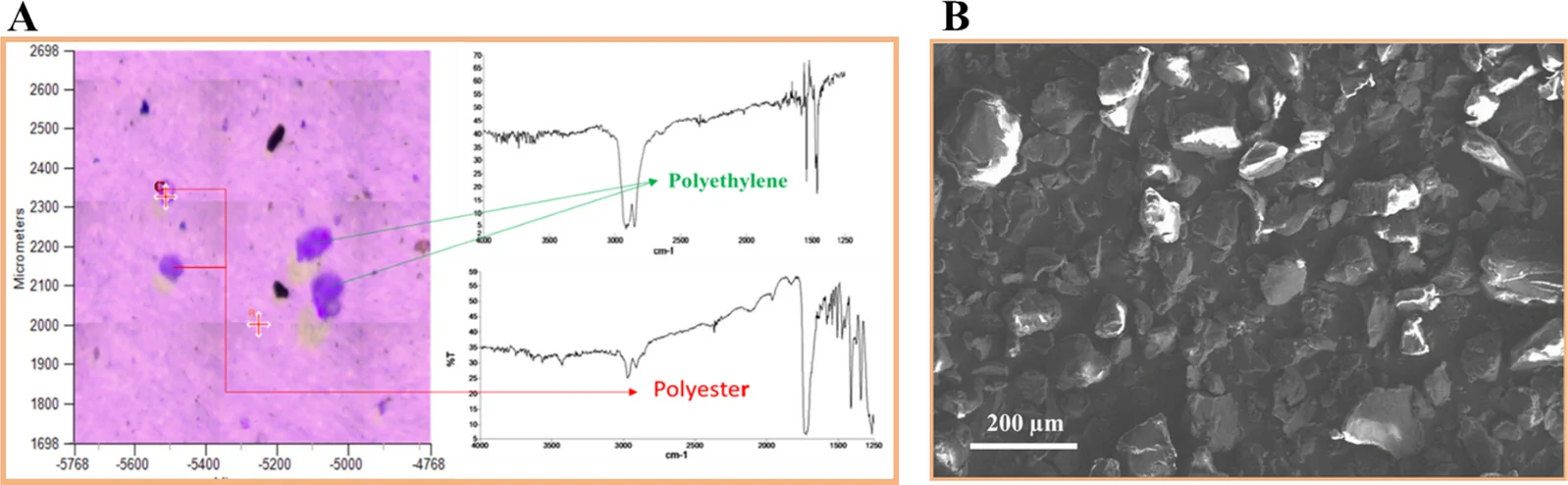
(**A**) The distribution and polymer identification of the MPs on anodisc filter. (**B**) SEM Image of MPs

In this study, raw laundry wastewater with an initial MPs concentration of 9,000–11,000 particles L⁻^1^ was used as the influent. PET and PE were identified as the dominant polymer types in the wastewater. The MPs concentration was substantially smaller than that reported by Bortot Coelho et al. (2025), who observed concentrations of (1.4 ± 0.7) × 10⁶ particles L⁻^1^ in laundry effluent, highlighting the considerable variability in MPs release due to differences in textile composition, washing conditions, and analytical methods. The resulting MPs displayed a broad size distribution, ranging from the nanometer scale to 200 µm (Fig. 4B). The distribution and polymer identification of the MPs before and after filtration were simultaneously characterized, as shown in Fig. 4A.

**Fig. 5:**
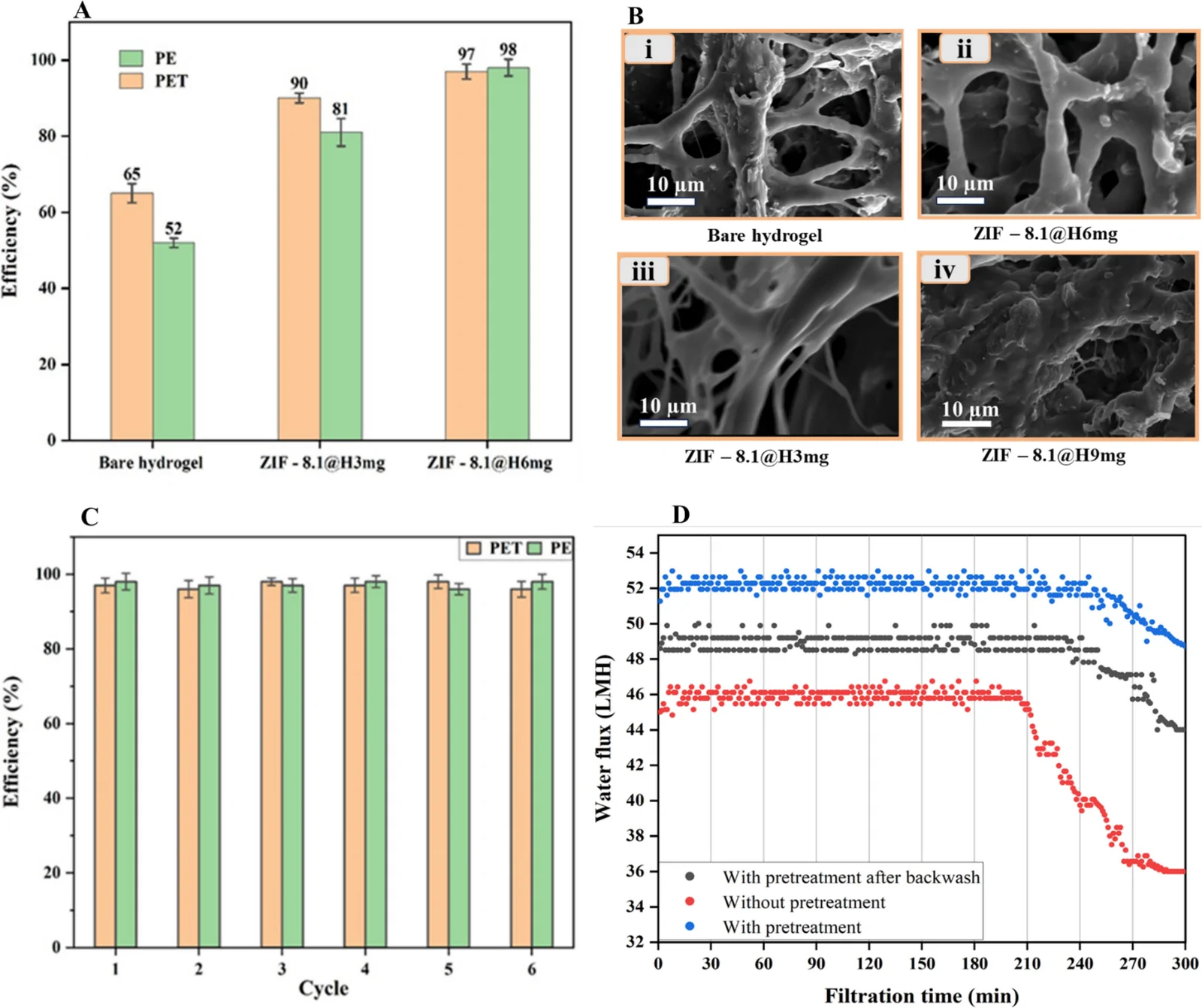
(**A**) The removal efficiency of 3 materials for PET and PE MPs. (**B**) The SEM images of hydrogel materials before filtration (**C**). The recycling performances of ZIF – 8.1@6 mg. (**D**). The water flux of ZIF—8.1@6 mg

The corresponding removal performance is presented in Fig. 5A. Overall, the ZIF-8-based hydrogel showed consistently higher PET removal performance than PE. The bare hydrogel showed the lowest performance (65 ± 2.5% for PET and 52 ± 1.2% for PE), which can be attributed to its negatively charged surface; since most MPs also carry negative charges, the electrostatic interactions are weak (Nguyen et al. 2021; Schneider et al. 2004). Upon incorporation of ZIF-8 (3 mg), the efficiency significantly increased to 90 ± 1.3% for PET and 81 ± 3.6% for PE. At higher loading (6 mg), among the tested materials, ZIF–8.1@H6mg exhibited the highest removal efficiency, achieving 97 ± 2% for PET and 98 ± 2.2% for PE. The effective performance is higher than that reported in a previous study using a metal–organic framework-based wood aerogel (85%) (You et al. 2021) and comparable to that of metal–organic framework-based foams (95%) (Chen et al. 2020).

**Fig. 6:**
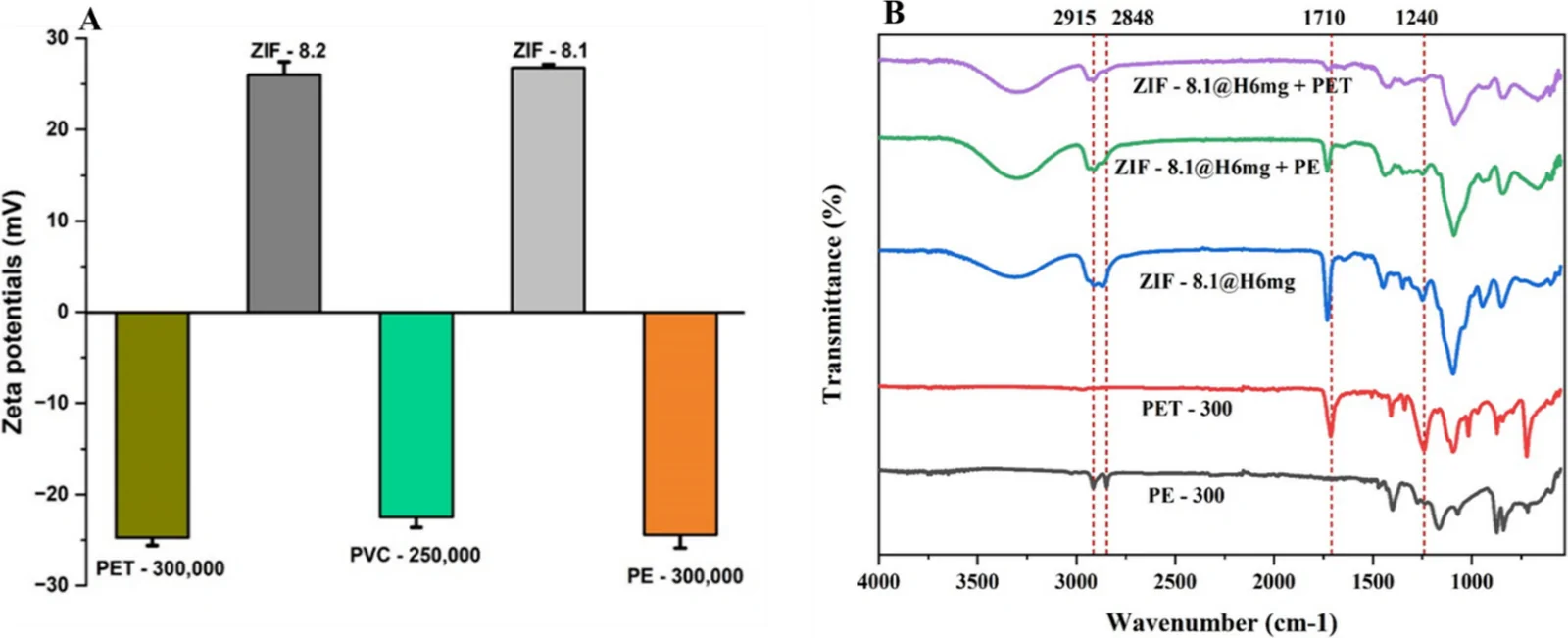
(**A**) The z potentials for various ZIF −8 and microplastics. (**B**). FTIR of ZIF-8–based hydrogel and MPs particles before and after the MPs removal process

The SEM images of the hydrogel composites are also consistent with the performance of each type of hydrogel composite. While the structure of ZIF–8.1@H3mg was relatively loose, the ZIF–8.1@H6mg sample showed nanocrystals dispersed on the hydrogel surface without disrupting the intrinsic pore structure of the bare hydrogel (Fig. 5Bi, ii, iii). Notably, when the ZIF-8.1 loading was further increased to 9 mg, wastewater could no longer permeate through the ZIF-8.1@9 mg hydrogel. This phenomenon may be attributed to excessive ZIF-8, which causes particle aggregation, thereby reducing porosity and adversely affecting the permeability of the hydrogel (Fig. 6B.iv). Subsequently, it can be concluded that the proportion of the MOF (ZIF–8.1) incorporated during the fabrication of the hydrogel composite plays a crucial role in determining the composite's MP removal efficiency. Thus, ZIF–8.1@H6mg is a promising candidate for subsequent recyclability and fouling experiments.

Recyclability is a key parameter in evaluating the durability of ZIF-8–based hydrogel materials. Among the tested samples, ZIF–8.1@H6mg, which exhibited the best performance (97 ± 2% for PET and 98 ± 1.1% for PE), was selected as the optimal platform to assess the adsorbent recycling behavior. The experimental results show that the material could be reused for repeated MPs removal while maintaining its high removal efficiency, with PET removal rates of 97 ± 2% in the first run, 98 ± 1.2% in the third run, and 96 ± 2.1% in the sixth run (Fig. 5C). Furthermore, the SEM image of the ZIF–8.1@H6mg sample collected after the sixth filtration run revealed the presence of nanoscale MPs (Fig. S7i), a feature that previous studies employing sand filtration or coagulation methods had not achieved (Gao et al. 2022; Talvitie et al. 2017).

To further assess the composite after repeated use, SEM analysis was conducted on the ZIF-8.1@H6mg sample following six filtration–regeneration cycles. As shown in Figure S7ii, nanocrystal-like features were still observable on the hydrogel surface after regeneration, suggesting that the composite’s overall morphology was largely preserved during repeated operation. This recyclability highlights not only the economic feasibility but also the environmental relevance of the material. Nevertheless, further studies involving a greater number of filtration–regeneration cycles are required to comprehensively evaluate the material’s long-term recyclability and durability.

Figure 5D illustrates the fouling behavior of the ZIF-8.1@H6mg hydrogel composite during the filtration of MP-rich laundry wastewater under three operating conditions: with pre-treatment, with pre-treatment followed by backwashing, and without pre-treatment. The data precisely identifies a critical fouling transition point after 200–210 min of continuous operation, particularly for the 'Without pretreatment' sample, which exhibited a rapid 21.7% flux decline (from ~ 46 to ~ 36 LMH) within the 300-min cycle. This acute decline in wastewater mirrors the rapid, irreversible fouling kinetic trends noted when trace MPs interact with secondary effluent organics (Pizzichetti et al. 2023). However, with pretreatment, the performance stabilized significantly, with only a 5.8% flux decline over the same period. This high-resolution quantification of flux preservation stands in stark contrast to the 9% to 54% flux declines reported by Hube et al. (2024) during the filtration of primary municipal wastewater laden with MPs. These results demonstrate that pretreatment effectively mitigates severe initial fouling by maintaining a more stable and higher operating flux compared to conventional systems treating real wastewater matrices.

An additional practical advantage of the developed ZIF-based hydrogel is that it can be freeze-dried and stored at ambient conditions without special preservation (Fig. S4). Furthermore, the hydrogel can be fabricated in different dimensions to accommodate various filtration column configurations, highlighting its potential for scale-up and practical water treatment applications. Regarding the cost analysis, the material preparation and operating costs of the lab-scale column were estimated to assess its economic viability for the removal of MPs from laundry effluent (Rai et al. 2023; Soni et al. 2020; Sorour et al. 2020; Yousef 2025). Based on the experimental data, it is assumed that the column is continuously operated for 1 year (365 days) at a filtration rate of 1.7 m h⁻^1^, and each hydrogel filter is used for up to 6 cycles, 4 h/cycle (24 h of operation) before being replaced with a new filter. Details about the calculation are presented in Table S2. It is important to note that the total annual cost consists of: (1) chemical cost for material fabrication; and (2) energy consumption for material preparation. As presented in Table S2, the total annual cost of the filtration column for microplastic removal from laundry wastewater was estimated at approximately 3.1 AUD, corresponding to the treatment cost of 0.3 AUD per cubic meter of laundry wastewater.

Furthermore, Table 2 below compares the performance of the hydrogel filtration material developed in this study with other reported technologies in terms of MPs removal efficiency and treatment cost. The results demonstrate that the MOF-derived hydrogel offers a promising and economically viable approach for efficient MPs removal, highlighting its potential as a sustainable alternative to existing technologies. Nevertheless, further comprehensive investigations are required to evaluate its long-term performance, scalability, and techno-economic feasibility for industrial-scale production and practical implementation.

**Table 2:** Comparison between this study and other technologies with regard to MPs removal efficiency and treatment cost

Technologies	MPs source	Removal efficiency (%)	Treatment cost (AUD/m^3^)	References
Electro Coagulation	Wastewater Treatment Facilities	99	2.2	(Elkhatib et al. 2021)
Electro coagulation/electro flotation hybrid process	Laundry Wastewater	98	1.9	(Akarsu and Deniz 2021)
Photocatalytic Membrane Reactor	Hospital Laundry Wastewater	96	2.6	(Bortot Coelho et al. 2025)
Fuller's Earth And Bagasse Ash Ceramic Membrane	Synthetic Wastewater	95—99.5	77.4	(Kolluru and Raja 2026)
MOF-Derived Hydrogel	Laundry Wastewater	97—98	0.3	***This study***

The results demonstrate that the MOF-derived hydrogel offers a promising and economically viable approach for efficient MPs removal, highlighting its potential as a sustainable alternative to existing technologies. Nevertheless, further comprehensive investigations are required to evaluate its long-term performance, scalability, and techno-economic feasibility for industrial-scale production and practical implementation.

### Microplastics removal mechanism

As noted above, a series of ZIF-8–based hydrogel materials containing uniformly distributed ZIF-8 nanocrystals was synthesized. To evaluate their MPs removal mechanism, the interactions between the hydrogel materials and MPs were firstly investigated using zeta potential measurements of the two types of ZIFs and MPs. The zeta potentials of ZIF-8–1 and ZIF-8–2 were measured as 26.80 ± 0.36 mV and 26.03 ± 1.40 mV, respectively. In contrast, most MPs exhibit negative surface charges (Zhou et al. 2021). In this study, the measured zeta potentials were − 24.73 ± 0.85 mV for PET (Molecular weight (Mw) = 300,000 g mol^−1^), − 24.43 ± 1.45 mV for PE (Mw = 300,000 g mol^−1^), and − 22.47 ± 1.17 mV for PVC (Mw = 250,000 g mol^−1^) (Fig. 6A). Consequently, the uniformly distributed ZIF-8 nanoparticles, carrying abundant positive charges, could facilitate strong electrostatic interactions with the negatively charged MPs.

Subsequently, infrared spectroscopy was applied to examine the interactions between the fabricated ZIF-8–based hydrogel and MPs particles before and after the MPs removal process, providing insights into their possible interaction mechanisms at the molecular level (Fig. 6B**).**

The spectrum of ZIF-8.1@H6mg exhibits the characteristic vibrational bands of ZIF-8, including the C = N stretching at ~ 1580 cm⁻^1^, imidazole ring vibrations in the range of 1140–990 cm⁻^1^, and the Zn–N band below 800 cm⁻^1^.

PET-300 displays strong ester-related absorptions, with a C = O stretching band at ~ 1710 cm⁻^1^, C–O stretching at ~ 1240 cm⁻^1^, and C–O–C at ~ 1100 cm⁻^1^, together with aromatic skeletal vibrations in the 1400–1000 cm⁻^1^ region (Fan et al. 2021). Upon adsorption of PET-300, the composite spectrum reveals overlapping of PET peaks (~ 1710, ~ 1240 cm⁻^1^) with those of ZIF-8, accompanied by slight intensity reductions. This suggests that adsorption could proceeds mainly through surface interactions such as hydrogen bonding and dipole–dipole interactions between the polar ester groups of PET and the C = N/N–H moieties of ZIF-8, in combination with micropore confinement (~ 1.16 nm).

In contrast, PE-300 exhibits a relatively simple spectrum dominated by C–H stretching bands at 2915 and 2848 cm⁻^1^ and C–H bending modes at 1470 and 720 cm⁻^1^, consistent with its nonpolar nature (Morgado et al. 2021). After filtration, the composite spectrum shows the emergence of C–H bands (~ 2915, ~ 2848 cm⁻^1^), albeit with weaker intensity than PET, reflecting weaker interactions. Given the absence of polar functional groups, PE adsorption could occur primarily via van der Waals forces and hydrophobic interactions, supplemented by mechanical entrapment within the porous ZIF-8 framework.

Comparatively, PET-300 appears to exhibit stronger adsorption, likely due to its polar functional groups enabling both weak chemical (physicochemical) and physical interactions. This behavior is consistent with previous findings, where a coagulation method was applied to remove PET MPs (Zhou et al. 2021; Luu et al. 2025). However, PE-300 is retained mainly through physisorption and mechanical trapping. The adsorption mechanisms are predominantly governed by physisorption rather than by covalent or coordination bond formation.

**Fig. 7:**
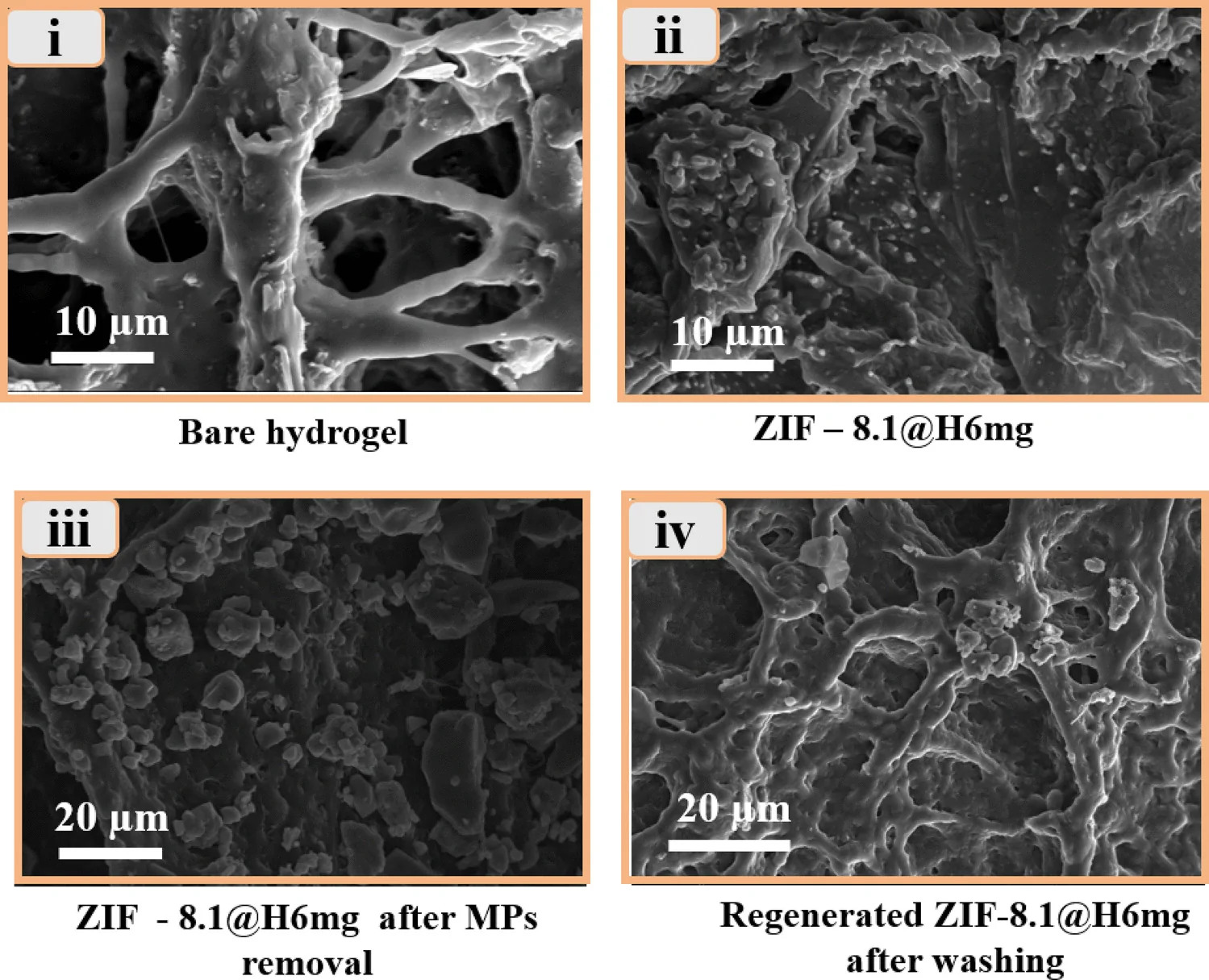
SEM images of (**i**) bare hydrogel, (**ii**) ZIF-8.1@H6mg composite hydrogel, (**iii**) ZIF-8.1@H6mg after MPs removal, and (**iv**) regenerated ZIF-8.1@H6mg after washing

The bare hydrogel exhibited a highly porous three-dimensional network with interconnected pores (Fig. 7i), which may facilitate water transport through the material. After ZIF-8.1 incorporation, numerous particulate features appeared on the hydrogel skeleton (Fig. 7ii), which are consistent with the presence of ZIF-8.1 nanocrystals. This is supported by the FTIR and XRD results which have been discussed previously. Following the MPs treatment, the hydrogel surface was covered by a substantial amount of deposited particles (Fig. 7iii), suggesting the accumulation of retained MPs during the filtration process. This behavior may be attributed to a combination of physical sieving within the porous structure and interactions between MPs and the ZIF-8.1-modified hydrogel, including electrostatic attraction, hydrogen bonding, and van der Waals forces. After washing, most of the deposited particles observed in Fig. 7iii were no longer evident (Fig. 7iv), while the particulate features associated with ZIF-8.1 remained visible on the hydrogel framework. These observations suggest that the composite maintained its structural integrity after regeneration, consistent with its stable performance across multiple reuse cycles.

In summary, the high MPs removal efficiency of ZIF-8.1@H6mg is likely attributed to the synergistic effects of the interconnected hydrogel network and the incorporated ZIF-8 nanoparticles. SEM observations suggest the accumulation of retained particles after filtration and the preservation of the material structure after regeneration, indicating good stability and reusability. The hydrogel network provides a physical filtration framework, while ZIF-8 nanoparticles are likely to enhance particle retention through electrostatic attraction and other interfacial interactions (Han et al. 2025). These findings suggest that the removal of MPs may involve a combination of filtration and particle–surface interactions rather than adsorption alone, which is consistent with recent studies reporting that MPs commonly associate with organic matter and microorganisms to form larger flocs in aquatic environments (Wu et al. 2024).

## Conclusion

In summary, a series of metal–organic framework-based hydrogels was successfully fabricated through a unique UV-initiated cross-linking method, providing a simple and efficient preparation method. The integration of stable ZIF-8 into the hydrogel matrix at an optimal ratio resulted in MOF–hydrogel composites with high uniformity and mechanical stability, which can be effectively applied for the removal of MPs across various types. Notably, the best-performing material, namely ZIF – 8.1@H6mg achieved high removal efficiencies of up to 97% for PET and 98% for PE in laundry wastewater containing a high concentration of MPs, while maintaining excellent recyclability over six reuse cycles. The MOF-based hydrogel ZIF-8.1@H6mg also operates stably for about 4 h, with pretreatment and backwashing further enhancing flux sustainability.

The properties of the ZIF-8 hydrogel composites, as well as the proposed mechanisms of MPs removal—such as the interpenetrating porous structure, zeta potential, and surface functional groups—were systematically investigated and discussed to elucidate their superior performance. Future studies will focus on scaling up the hydrogel materials and filtration columns for large-scale applications while further reducing production costs. Further work will also investigate the effects of operational parameters such as pH, temperature, and influent MPs concentration to optimise treatment performance and improve process applicability under varying wastewater conditions. Advanced characterisation techniques such as X-ray Photoelectron Spectroscopy (XPS) and Raman spectroscopy, are proposed for future studies to further verify the proposed MPs removal mechanisms. These ZIF-based hydrogel materials, with their outstanding MPs removal efficiency, hold great promise as sustainable alternatives for addressing the increasingly severe issue of MPs pollution.

## Supplementary Information

Below is the link to the electronic supplementary material.

ESM 1 (DOCX 8.47 MB)

## Data Availability

The datasets used and analysed during the current study are available from the corresponding author on reasonable request.
