# Supplementary material for: Synthesis of metal organic framework-based hydrogel for microplastics removal from aquatic environments
Source: Environ Sci Pollut Res Int. 2026 Sep 1;33(28):14165–77. doi: 10.1007/s11356-026-38156-2 (PMC13582124; doi:10.1007/s11356-026-38156-2)
Supplement: Supplementary file 1 [file 11356_2026_38156_MOESM1_ESM.docx]

***Supplementary data for***

**Synthesis of Metal Organic Framework-based Hydrogel for Microplastics Removal from Aquatic Environments**

*Thi Trang Luu^1,2^, Shudi Mao^1^, Dai Quyet Truong^1^, Yihan Shi^1^, Thi Ngoc Oanh Duon^1^, Qiang Fu^1^, Van Manh Do^3^, Saravanamuthu Vigneswaran^1^, Tien Vinh Nguyen^1^**

^1^Faculty of Engineering and IT, University of Technology Sydney (UTS), Sydney, NSW 2007, Australia

^2^Faculty of Urban Infrastructure and Environment Engineering, Hanoi Architectural University, Hanoi, 100000, Vietnam

^3^Institute of Science and Technology for Energy and Environment, Vietnam Academy of Science and Technology (VAST), Hanoi, 100000, Vietnam

*Correspondence: [tien.nguyen@uts.edu.au](mailto:tien.nguyen@uts.edu.au)

**Table**

**Table S1** Quantitative comparison of the most intense diffraction peak of ZIF-8.1 and ZIF-8.2.

| **Sample** | **Peak position (2Ɵ/ ^o^)** | **Peak intensity (a.u.)** | **FWHM (^o^)** |
| --- | --- | --- | --- |
| ZIF-8.1 | 17.66 | 18.38 | 0.18 |
| ZIF-8.2 | 12.66 | 12.73 | 0.42 |

**Table S2** Cost estimation for the lab-scale filtration column (Filtration rate: 1.7 m h⁻¹; Each hydrogel filter: used for 6 cycles, 4 hours/cycle before being replaced; Total volume of filtrated water/year:10.5 m^3^)

| **(1) Chemical cost for 40g product (1 year operation)** | **Price (AUD)** |  | **(2) Energy consumption for material fabrication (0.35AUD/kWh)** | | **Price (AUD)** |  |
| --- | --- | --- | --- | --- | --- | --- |
| ZIF preparation | 0.80 |  | ZIF preparation | | 0.01 |  |
| 10 wt% PVA solution | 0.66 |  | 10 wt% PVA solution | | 0.01 |  |
| PEGDA | 0.31 |  | Hydrogel mixture | | 0.01 |  |
| LAP | 0.01 |  | UV irradiation (for 365 pieces) | | 1.28 |  |
| **Total (1)** | **1.78** |  | **Total (2)** | **1.31** | |  |
| **Treatment cost/annual: (1) + (2): 3.09 AUD** | | | | | | |
| **Treatment cost/m^3^ treated water: 0.3 AUD** | | | | | | |

**Figure**

**Fig. S1.** Preparing the laundry wastewater with high strength MPs

**Fig. S2.** Microplastics analysis using the PerkinElmer Spectrum 3 FTIR spectrometer equipped with a Spotlight 200i microscope (PerkinElmer, USA) ng

**Fig. S3**. XRD patterns of (a) ZIF-8.1 and (b) ZIF-8.2 synthesized under different HMIM:Zn molar ratios. The original diffraction patterns are presented separately for clarity, while the overlaid comparison is shown in Figure 1 of the main manuscript.

**Fig. S4.** Photographs of the ZIF-based hydrogel before and after freeze-drying. The freeze-dried material can be stored under ambient conditions without special preservation

.

**Fig. S5.** Photograph of the laboratory-scale fixed-bed column setup employed to evaluate the microplastic removal performance of the ZIF-8/hydrogel composite under continuous-flow condition

|  |  |
| --- | --- |
|  |  |

**Fig. S6.** SEM-EDS elemental mapping of ZIF-8.1@H6mg hydrogel composite, illustrating the spatial distribution of the primary elements carbon (C), oxygen (O), and zinc (Zn) within the material.

**Fig. S7(i).** SEM image of the ZIF-8.1@H6mg sample after the sixth filtration cycle, showing retained nanoscale microplastic particles on the hydrogel surface.

(**ii**). SEM image of ZIF-8.1@H6mg after the sixth filtration cycle and regeneration, showing the persistence of ZIF-8 nanocrystals on the hydrogel surface.
